# Supplementary material for: Hemodynamics in chronic pain: A pathway to multi-modal health risks
Source: PLoS One. 2024 Dec 10;19(12):e0315341. doi: 10.1371/journal.pone.0315341 (PMC11630596; doi:10.1371/journal.pone.0315341)
Supplement: S1 Materials — (PDF) [file pone.0315341.s001.pdf]

S Materials

Hemodynamics in chronic pain: A pathway to multi-modal health risks

Dmitry M. Davydov [1*], MD, PhD, Dr. med. hab.

Carmen M. Galvez-Sánchez [2], PhD

Gustavo A. Reyes del Paso [3], PhD

[1] María Zambrano senior scholar, University of Jaén, Jaén, Spain

[2] Department of Personality, Evaluation and Psychological Treatment, University of Murcia, Murcia, Spain

[3] Department of Psychology, University of Jaén, Jaén, Spain

* Corresponding author

Email: d.m.davydov@gmail.com (DMD)

The Supporting Information encompass:

S Materials.I. Findings on within-subject effects on hemodynamics across sessions.

S Materials.II. Detailed examination of posture-induced gravity-related effects on body fluid translocations.

S Materials.III. In-depth discussion of different theories explaining rapid transient vasodilation upon standing, framed within the current study's findings.

S Materials.IV. Alternative interpretations of imbalanced cardiac effects on hemodynamics in patients.

S Materials.V. Extended discourse on the total blood volume deficit as a potential primary cause underlying the pain-o-metric patterns identified in hemodynamic and cardiovascular responses to orthostatic and clinostatic challenges.

S Materials.VI. Thorough exploration of the consistency of current findings with those of previous studies.

S Materials.VII. Exploration of various strategies for rebalancing central and peripheral mechanisms to restore body water and blood volume redistribution in response to orthostatic and clinostatic challenges (this section also includes perspectives on the development of new technologies for managing and preventing pain syndromes).

S Materials.VIII. Insights into the application of the data-driven analytical technique employed in the study, particularly in relation to the regulatory mechanisms underlying hemodynamic and cardiovascular fluctuations during steady states.

S Materials.IX. The electrode placements for electrocardiogram (ECG) and impedance cardiogram (ICG) acquisitions adhered to the study's established protocols for hemodynamic and cardiovascular measurements.

S Materials.I. Findings on within-subject effects on hemodynamics across sessions.

While chronic pain may influence periodic and aperiodic clino-orthostatic responses in hemodynamic and cardiovascular (CV) processes between sessions, this study adopted a conservative approach by analyzing the effects of repeated clino-orthostatic challenges as a general factor. Thus, the analysis did not account for the differences between fibromyalgia (FM) and healthy samples as an additional influencing factor, leaving a more detailed examination of interaction effects for future research.

Between-session (within-subject) effects, as illustrated in Figs 1S-7S below, were noted in several transient hemodynamic and CV measures. Specifically, there was an increase in cardiac output (CO) accompanied by a rise in heart rate (HR) and deeper drops in systemic vascular resistance (SVR), systolic blood pressure (SBP), and diastolic blood pressure (DBP) during session 2 compared to session 1 in response to gravity-related hemodynamic challenges. These findings suggest that the mechanisms underlying these short-term responses may be modifiable and sensitive to training or conditioning, potentially enhancing physiological resilience to gravitational stress and promoting body resilience against chronic pain and its associated risk factors for hypertension [1–5]. Moreover, as preload-regulating mechanisms, including reactive vasodilation and skeletal-muscle contractions along with baroreceptor sensitivity specifically to their loads, were found to be sensitive to voluntary and involuntary conditioning manipulations, including repeat physical exercise and electrical stimulations [1–4,6], they may be suggested for further investigation. In particular, in individuals with their malfunction, as in the FM patients, interventions could target increasing their efficiency in response to clino-orthostatic challenges

as a probable mechanism for protecting (increasing resiliency) against this pathophysiological pathway to hypertension during chronic pain syndrome progression.

Additional between-session (within-subject) effects, as shown in Figs 1S-7S, revealed notable long-lasting hemodynamic and CV responses. Stroke volume (SV) and cardiac contractility (measured by pre-ejection period, or PEP) remained nearly constant, while HR decreased, leading to reduced CO. This was accompanied by an increase in SVR and more stable SBP and DBP trends during standing. However, in session 2, SBP and DBP fluctuations were twice as high—7 and 4 cycles per minute, respectively, according to effective degrees of freedom (edf) values—compared to session 1, which showed fluctuations of 3 and 2 cycles per minute, respectively. The edf measure represents the actual turning points of hemodynamic and CV curves identified through a data-driven analytical technique over the period of analysis, which in this case was 600 seconds.

These findings demonstrated that the changes in CO and SVR between sessions were precisely cross-compensated, as evidenced by their reciprocal opposing shifts, with similar edf values transferring to 4 cycles per minute in session 1 and 7 cycles per minute in session 2 for both measures. Cardiac contractility (inotropy) remained nearly consistent in levels, trends, and fluctuations (around 6 cycles per minute, according to edf values) across sessions. This suggests a finely tuned balance between preload and afterload processes, consistently observed, likely reflecting a combined hemodynamic and CV response to mild dehydration influencing the body's short-term hydration balance between sessions.

Support for this mild between-session dehydration is also provided by changes in the edf measure. These changes reflected an increase in HR fluctuations in the high-frequency band (i.e.,

within the respiratory rate range), rising from approximately 16 cycles per minute in session 1 to 18 cycles per minute in session 2. A detailed explanation of this mechanism is provided in S Materials VIII, where it is linked to respiratory baroreflex activities that respond to BP changes by adjusting respiration rate and depth, thereby establishing venous pressure gradients to regulate venous return and cardiac preload. The increase in HR fluctuation suggests that by session 2, the CV system required more venous blood to be recruited from unstressed reserves to the arterial circulation to maintain BP within its homeostatic range. This adjustment was effective, as SV levels remained nearly identical between sessions, with fluctuations close in range—approximately 4 cycles per minute in session 1 and 5 cycles per minute in session 2, according to edf values.

These findings, which incorporate both aperiodic and periodic fluctuations in various hemodynamic and CV processes, should enhance our understanding of their interactions as a unified mechanism with modifiable responses to repeated similar challenges.

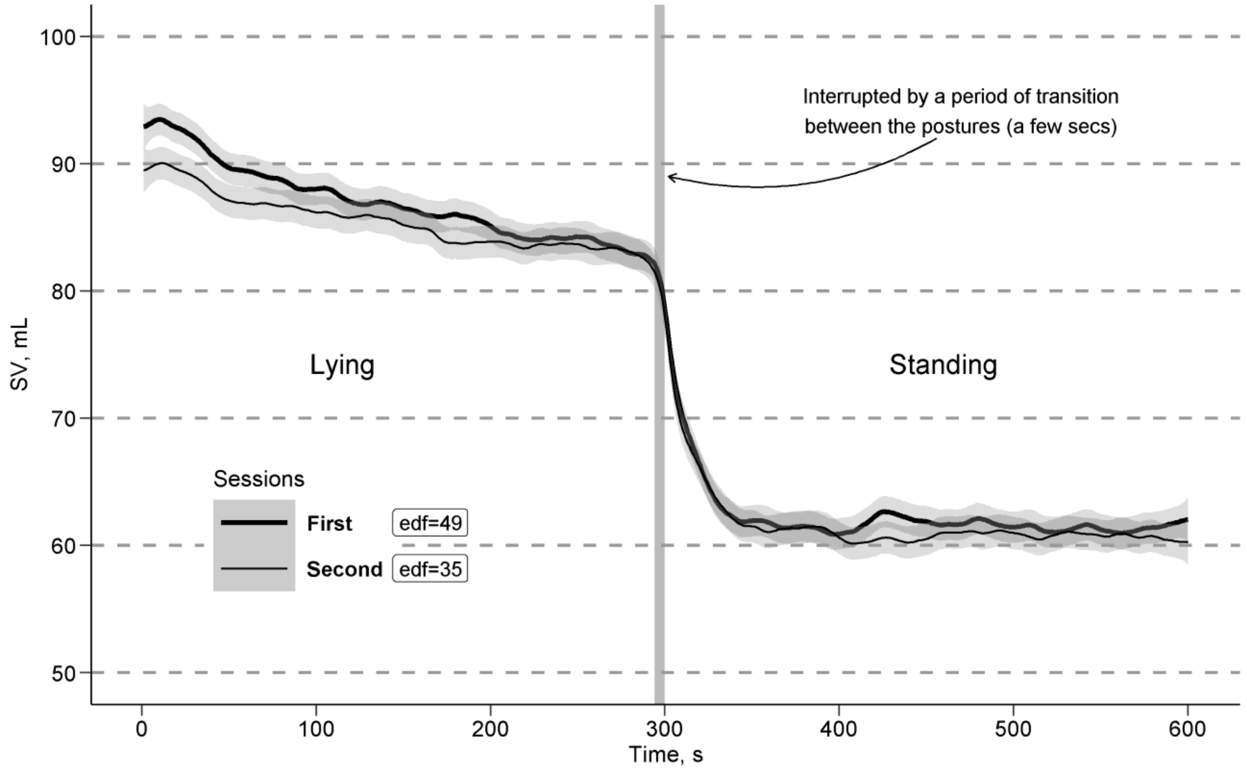

S1 Fig. Estimated time effects on stroke volume (SV) curves during the clino- and ortho-static challenges. The effects are averaged per Sessions 1 (bold line) and 2 (thin line) of the total sample of fibromyalgia and control groups, with approximate 95% confidence intervals (gray shading around the curves) and effective degrees of freedom or curve 'actual' turning points (edf).

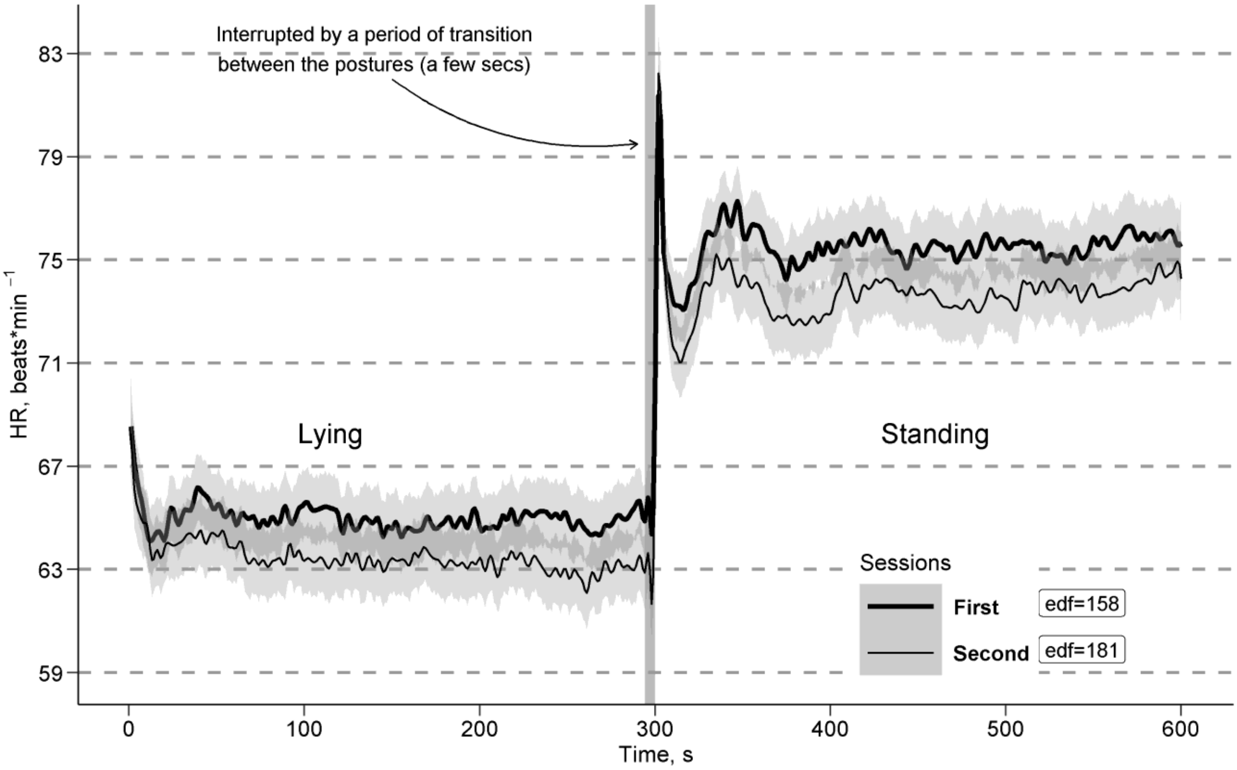

S2 Fig. Estimated time effects on heart rate (HR) curves during the clino- and ortho-static challenges. The effects are averaged per Sessions 1 (bold line) and 2 (thin line) of the total sample of fibromyalgia and control groups, with approximate 95% confidence intervals (gray shading around the curves) and effective degrees of freedom or curve 'actual' turning points (edf).

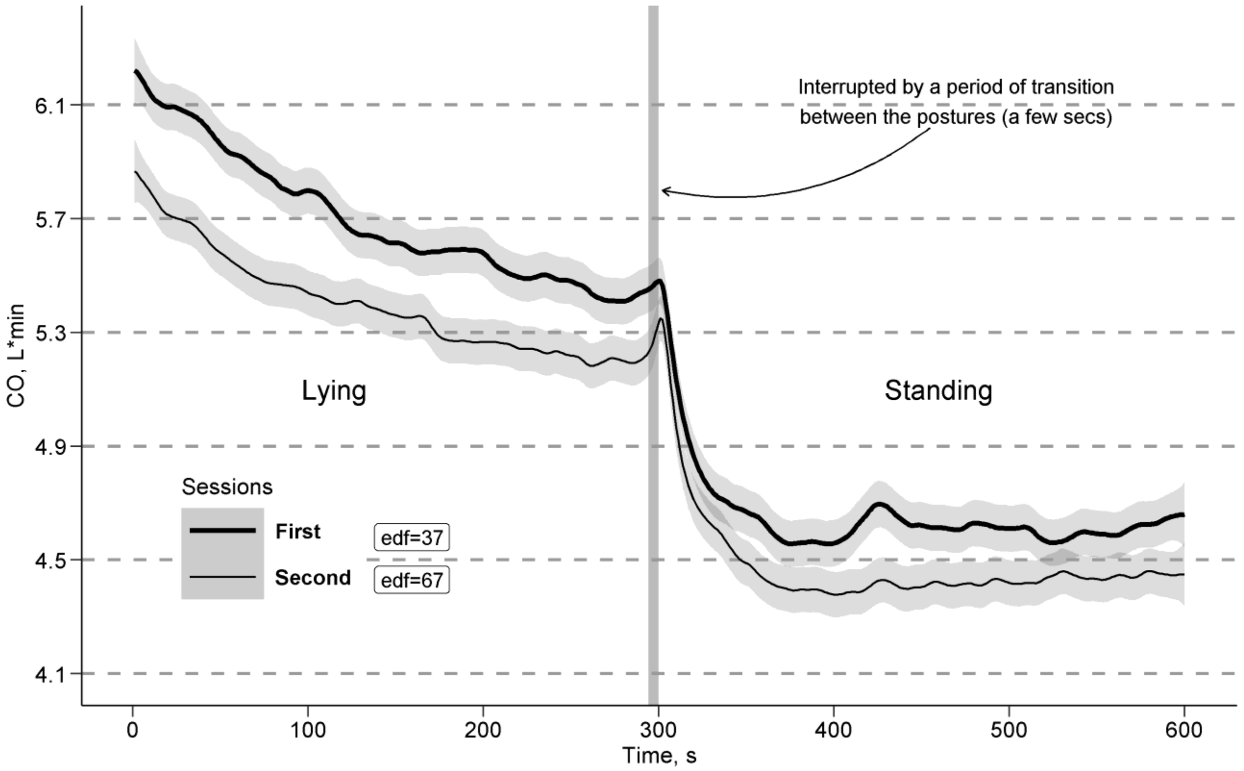

S3 Fig. Estimated time effects on cardiac output (CO) curves during the clino- and ortho-static challenges. The effects are averaged per Sessions 1 (bold line) and 2 (thin line) of the total sample of fibromyalgia and control groups, with approximate 95% confidence intervals (gray shading around the curves) and effective degrees of freedom or curve 'actual' turning points (edf).

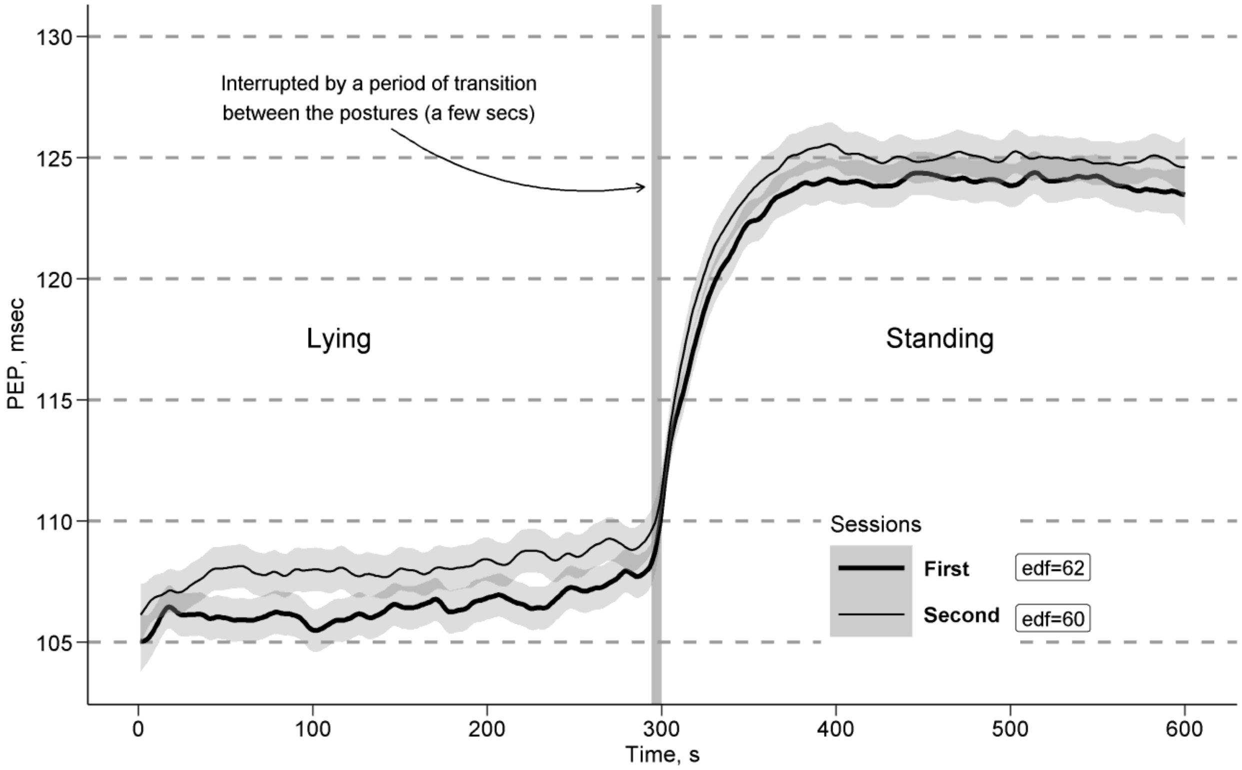

S4 Fig. Estimated time effects on pre-ejection period (PEP) curves during the clino- and ortho-static challenges. The effects are averaged per Sessions 1 (bold line) and 2 (thin line) of the total sample of fibromyalgia and control groups, with approximate 95% confidence intervals (gray shading around the curves) and effective degrees of freedom or curve 'actual' turning points (edf).

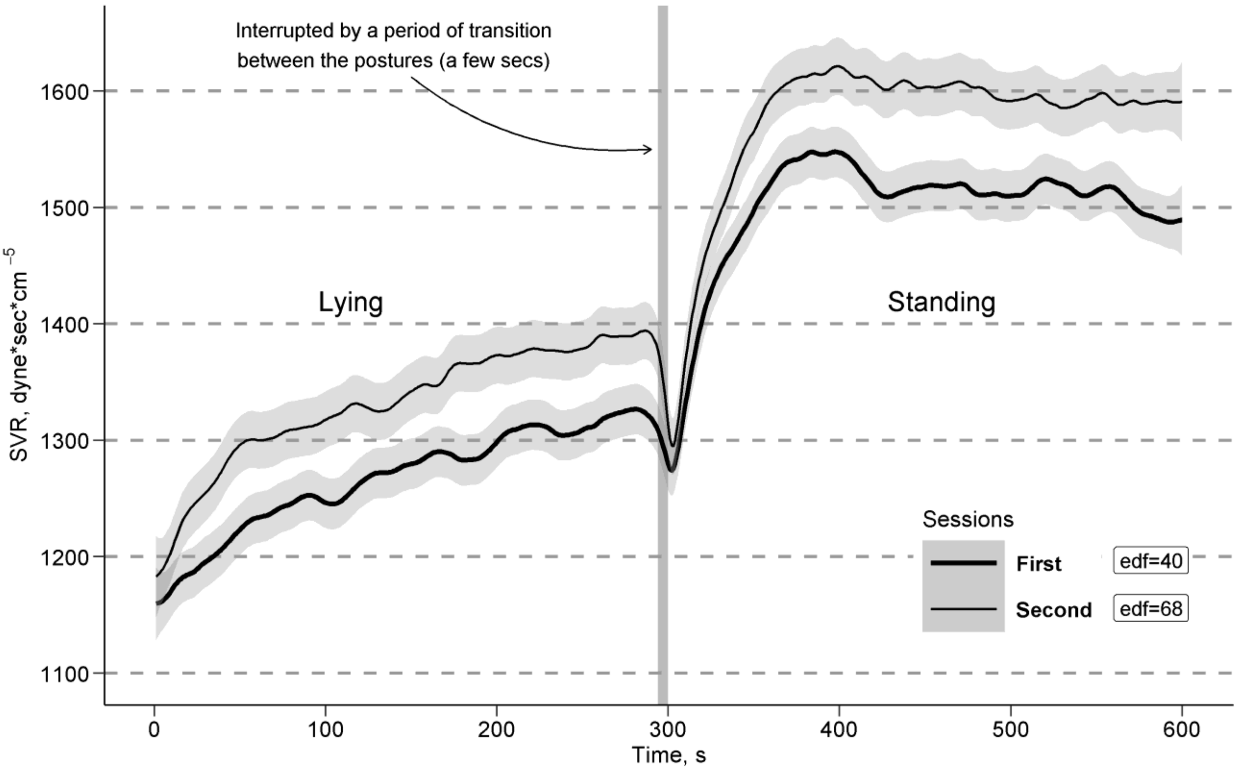

S5 Fig. Estimated time effects on systemic vascular resistance (SVR) curves during the clino- and ortho-static challenges. The effects are averaged per Sessions 1 (bold line) and 2 (thin line) of the total sample of fibromyalgia and control groups, with approximate 95% confidence intervals (gray shading around the curves) and effective degrees of freedom or curve 'actual' turning points (edf).

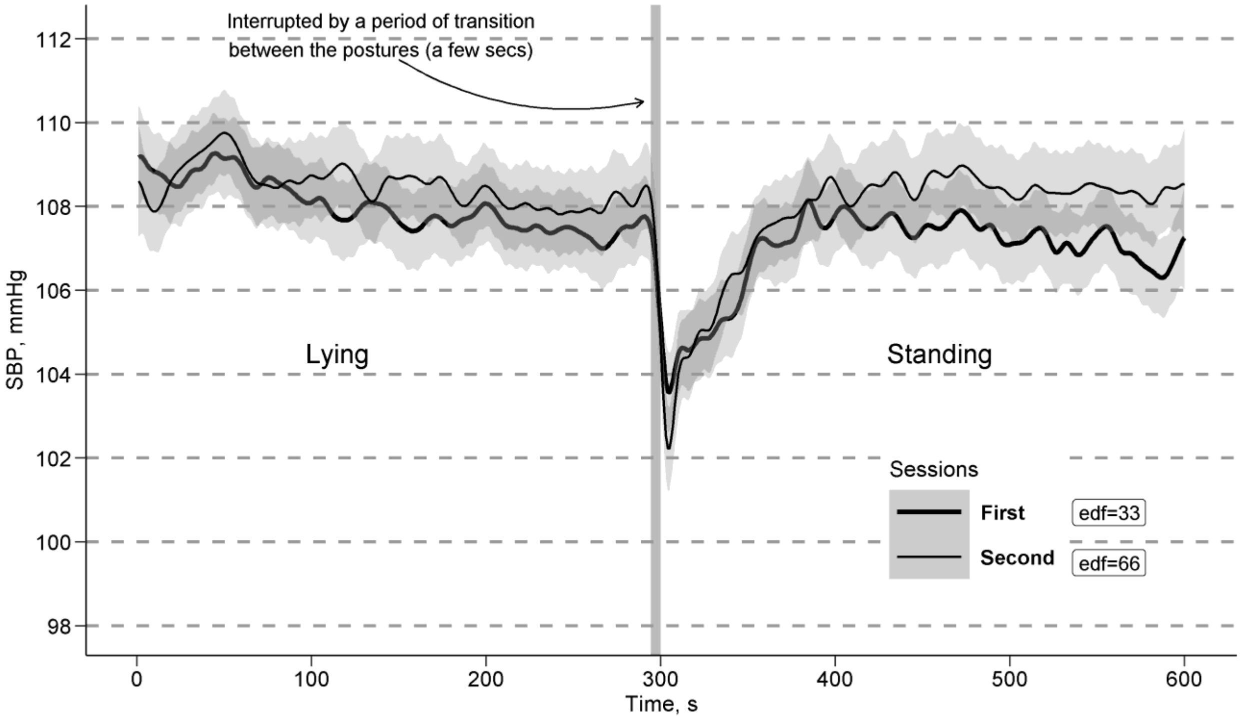

S6 Fig. Estimated time effects on systolic blood pressure (SBP) curves during the clino- and ortho-static challenges. The effects are averaged per Sessions 1 (bold line) and 2 (thin line) of the total sample of fibromyalgia and control groups, with approximate 95% confidence intervals (gray shading around the curves) and effective degrees of freedom or curve 'actual' turning points (edf).

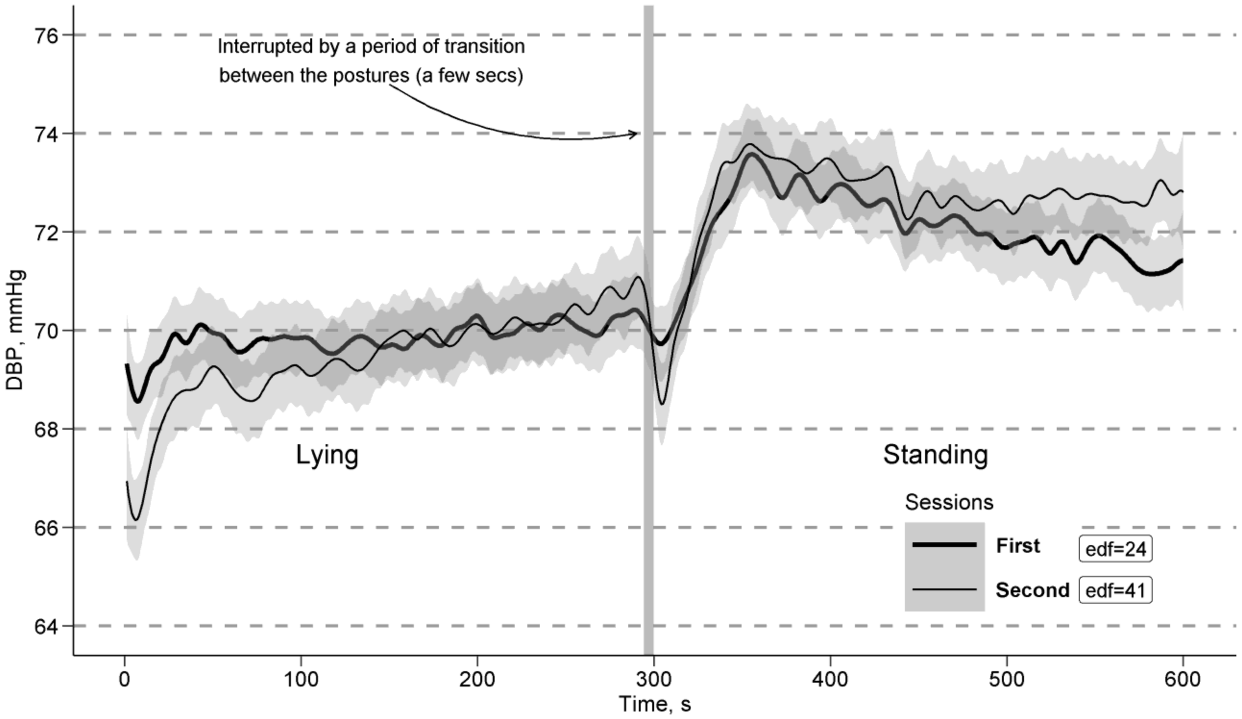

S7 Fig. Estimated time effects on diastolic blood pressure (DBP) curves during the clino- and ortho-static challenges. The effects are averaged per Sessions 1 (bold line) and 2 (thin line) of the total sample of fibromyalgia and control groups, with approximate 95% confidence intervals (gray shading around the curves) and effective degrees of freedom or curve 'actual' turning points (edf).

S Materials.II. Detailed examination of posture-induced gravity-related effects on body fluid translocations.

Previous studies have shown that clino- and ortho-static challenges prompt significant blood shifts of 500 to 1000 mL between upper and lower body parts due to gravitational forces. This results in a corresponding gain or loss of plasma volume (approximately 14% in healthy individuals, primarily within the initial 10 minutes of positional change) between tissues and blood circulation [7–12]. In response, the body utilizes mechanisms such as sodium and water deposition and release in muscles and skin derma, along with diuresis and fluid retention by the kidneys, to regulate effective circulating blood volume amidst the gravity-induced translocation of blood volume between peripheral and central organs and tissues [13,14].

As a result, these posture-induced gravity-related blood translocations, coupled with re- and de-hydration processes, cause fluctuations in venous return to the heart, leading to changes in cardiac preload and stroke volume (SV), which serves as a surrogate measure of end-diastolic blood volume. These changes subsequently impact arterial blood pressure (BP) and cerebral blood flow. To counteract these effects, the body initiates compensatory reflexes involving regulation of cardiac inotropy (contractility) and chronotropy (heart rate), along with systemic vascular capacity (vasoconstriction or vasodilation). These mechanisms are governed by various receptors that regulate the parasympathetic and sympathetic nervous systems, as well as the renin-angiotensin system and aldosterone release, serving as physiological resilience and adaptation mechanisms [15–17].

S Materials.III. In-depth discussion of different theories explaining rapid transient vasodilation upon standing, framed within the current study's findings.

Some researchers argue that the rapid transient vasodilation observed upon standing is a result of the formula used to calculate systemic vascular resistance (SVR), which may not accurately reflect actual vasodilation [11]. According to this perspective, the transient orthostatic vasodilation in healthy individuals occurs because of the continuation of vasodilation from the supine position to the early standing phase, before effective orthostatic vasoconstriction takes place. This initial drop in vascular resistance is seen as indicative of passive arterial inflow to the lower body, persisting from the supine position into early standing until lower body veins are refilled, temporarily suspending orthostatic vasoconstriction in response to gravity [11,18]. However, this viewpoint contradicts the notion that the strong initial transient systemic vasodilation observed during standing is a distinct event initiated by signals from systemic pulmonary artery mechanoreceptors and the release of peripheral autoregulatory agents, such as Adenosine Triphosphate (ATP), along with effective abdominal and skeletal muscle contractions [17,19–23].

According to the perspective that the initial transient vasodilation upon standing is not a genuine orthostatic-related change but rather a calculated phenomenon stemming from residual clinostatic vasodilation (a so-called ‘virtual’ orthostatic vascular conductance [24]), fibromyalgia (FM) patients would be expected to exhibit a more pronounced residual vasodilation immediately after standing compared to healthy individuals, reflecting their greater real vasodilation during the clinostatic period. Contrary to this assumption, the present study found

that healthy participants, rather than FM patients, exhibited a more significant initial orthostatic vasodilation. Thus, the findings of the present study suggest that FM patients may lack active changes in peripheral vessel diameters to increase vascular conductance shortly after standing, in contrast to healthy participants for whom such changes persist until the initiation of vasoconstriction reflexes.

Furthermore, the concept of ‘virtual conductance’ has been based on findings from passive head-up tilting experiments [24], rather than active standing, which diminishes its applicability to the active standing challenge and introduces discrepancies between responses observed in passive and active orthostatic probes, as noted in both the current and prior studies [12,21,23,25]. Additionally, if the ‘virtual conductance’ concept was accurate, one would expect more effective compensation for the effects of gravity by initial transient cardiovascular (CV) responses in FM patients compared to healthy participants, which contradicts observations. Adjustment for participants' height in the present study (i.e., for the difference in the initial pressure gradient between participants [11]) did not eliminate the initial transient vasodilation response in the healthy group, nor its statistical significance compared to FM patients, further undermining the ‘virtual conductance’ hypothesis.

Other researchers have also found that the hydrostatic pressure gradient alone, as proposed in the 'virtual conductance' concept, can only explain a small portion, if any, of the observed increase in blood flow during active standing [23]. They attribute this increase instead to actual rapid peripheral vasodilation triggered by peripheral vessel autoregulation mechanisms, which are determined by skeletal muscle contractions. Indeed, previous studies have shown that resistance vessels in skeletal muscle can dilate shortly (with a peak approximately three to four cardiac cycles) after a brief (as little as 0.3 s) muscle contraction release [23], a timing that aligns with

the findings of the present study. Therefore, these considerations and findings support the notion that the initial orthostatic vasodilation mechanism represents the actual reflexive or adaptive response to active standing, elucidating the differences in CV responses between healthy participants and FM patients.

S Materials.IV. Alternative interpretations of imbalanced cardiac effects on hemodynamics in patients.

As an alternative explanation for the disparity in baroreflex sensitivity, the reduced initial transient heart rate (HR) increase observed in fibromyalgia (FM) patients compared to healthy participants could be attributed to imbalanced cardiac afterload effects on hemodynamics. This imbalance was evident in the gradual monophasic rise of systemic vascular resistance (SVR) after standing in contrast to the biphasic response (drop and rise) of SVR observed in healthy participants. Consequently, the higher initial transient HR increase in healthy participants may be triggered by transient initial orthostatic vasodilation, thus negating the need to solely attribute the difference to impaired baroreflex sensitivity in FM patients. Previous research has demonstrated that immediate and transient orthostatic vasodilation is primarily observed during active standing rather than passive head-up tilting [11,17,19–22,26]. This response may be triggered by a combination of compensatory systemic and regional autoregulatory mechanisms, including low-pressure cardiopulmonary mechanosensitive receptors and the release of Adenosine Triphosphate (ATP) from skeletal muscle cells and erythrocytes. These mechanisms help recruit unstressed blood volume reserves from the mesentery and other organs during active contraction of abdominal and extremity muscles. These mechanisms regulate cardiac activity, specifically increasing preload, to counterbalance central exaggerated α-adrenergic vasoconstrictor effects, thereby stabilizing blood pressure and blood flow. This physiological response helps prevent hypoperfusion and decreased oxygen supply to the brain and peripheral tissues, potentially mitigating neuroinflammation, pain sensitization, and neuropathy, particularly in emergency hypovolemic or dehydration situations such as orthostatic stress. Unlike the initial transient vasodilation response to orthostatic stress, high orthostatic vasoconstriction as a prolonged steady state in response to hypovolemia was found to be similar during both active (standing)

and passive (head-up tilting) procedures. Other blood volume expansion mechanisms, such as water and electrolyte retention in the kidneys and fluid release from the skin, may be activated to counterbalance or reduce vasoconstriction if unstressed blood volume reserves in the mesentery and other organs are insufficient. These mechanisms are regulated by atrial mechanoreceptors that detect changes in blood volume [17,21,27–29].

S Materials.V. Extended discourse on the total blood volume deficit as a potential primary cause underlying the pain-o-metric patterns identified in hemodynamic and cardiovascular responses to orthostatic and clinostatic challenges.

This study evaluated initial rapid physiological reactions and their subsequent return to longer-term steady states in healthy participants and fibromyalgia (FM) patients. The analysis incorporated various hemodynamic and cardiovascular (CV) parameters to assess the response across both short- and long-term timeframes during a repeating complex clino-orthostatic challenge by treating them as parts of a unified body mechanism.

According to previous linear model studies [7,8,10,30], an orthostatic decrease in venous return and the resulting drop in stroke volume (SV) should lead to a reduction in systemic vascular capacity via peripheral vasoconstriction, helping to maintain blood pressure (BP) within the homeostatic range. This is in contrast to clinostatic conditions, where an increase in venous return and a subsequent rise in SV stimulate systemic vasodilation, leading to vascular capacity expansion. These mechanisms—vasoconstriction and vasodilation—are regulated through distinct pathways (renin-angiotensin and sympathetic activation or inhibition, respectively) to balance the cardiac volume-related effects of orthostatic dehydration and clinostatic rehydration.

The present study revealed that the orthostatic vascular constriction and clinostatic vascular relaxation, as indicated by systemic vascular resistance (SVR), were more pronounced in the patient group compared to healthy participants, especially in the initial few minutes following

transitions between postures. After an initial drop, vascular resistance in response to the clinostatic challenge increased more rapidly to its steady state in healthy participants, suggesting faster recovery, whereas in the patient group, recovery was delayed. Conversely, in response to the orthostatic challenge, the patient group showed an earlier rise in vascular resistance toward its steady state compared to healthy participants, who experienced a brief drop in resistance just after standing—likely indicating active orthostatic vasodilation—before resistance began to increase toward its steady state.

In healthy individuals, gravity-induced redistribution or translocation of body fluids leads to rapid central rehydration or dehydration, initially compensated through swift cardiac chronotropic responses—clinostatic heart rate (HR) deceleration and orthostatic HR acceleration, respectively—likely governed by reciprocal parasympathetic and sympathetic reactivity through cardiac baroreflex mechanisms. This is accompanied by a rapid response in vascular tone—clinostatic vasoconstriction or orthostatic vasodilation—possibly driven by cardiopulmonary volume-sensing reflexes and peripheral Adenosine Triphosphate (ATP) activity. These initial, quick CV responses to stabilize central body blood volume are further supported by changes in vascular tone—decreased during clinostatic phases or increased during orthostatic phases—and corresponding shifts in cardiac contractility to stabilize BP, which are likely regulated by slower and delayed renin-angiotensin and sympathetic nervous system responses. Thus, in normal conditions, these two successive early and later stages of the primary mechanisms of hemodynamic regulation in response to clino-orthostatic challenges work together to stabilize central blood volume and maintain consistent BP, thereby ensuring uninterrupted and efficient blood flow to the brain and peripheral tissues and supplying them with oxygen and nutrients appropriate to their metabolic demands.

In contrast, in FM patients, some of the primary compensatory mechanisms are either absent, disrupted, or replaced. For example, along with a stronger baroreceptor reflex indicated by a pronounced transient tachycardia, healthy individuals typically rely on a preload-based mechanism for cardiac contractility regulation where cardiac workload adjusts to fluctuations in venous return caused by the active relaxation and contraction of abdominal and skeletal muscles, which redistribute blood volume during lying and standing. In FM patients, however, the preload-based mechanism is replaced by an afterload-based mechanism, where cardiac contractility responds to changes in systemic vascular tone with less influence from venous return, due to reduced blood volume reserves for redistribution in response to gravity-induced challenges during lying and standing. This shift is also associated with a weaker baroreceptor reflex, evidenced by a less pronounced transient tachycardia. Additionally, while both groups experienced a gradual decrease in clinostatic SV after assuming the posture, the slope was steeper in the patient group, indicating a deficit in effective blood volume. Furthermore, the FM group lacked specific rapid orthostatic responses, such as an increase in cardiac output (CO). Instead of the biphasic pattern seen in the healthy group—with an initial brief overshoot followed by a decrease—the FM group exhibited a monophasic pattern with an immediate CO decline, further demonstrating a deficit in effective blood volume.

Thus, in FM patients, the alternative mechanisms of hemodynamic regulation in response to clino-orthostatic challenges maintain BP consistency without central blood volume stabilization, which leads to an uninterrupted but inefficient blood flow to the brain and peripheral tissues and oxygen and nutrients insufficient to meet their metabolic demands.

S Materials.VI. Thorough exploration of the consistency of current findings with those of previous studies.

In this study, most of the initial rapid hemodynamic and cardiovascular (CV) reactions and subsequent restoration to longer-term hemodynamic and CV steady states in healthy participants, in response to active orthostatic (standing) posture, were analyzed across various metrics and both short and long timeframes of a repeating complex clino-orthostatic challenge. Using data-driven non-linear models, the results aligned with earlier studies that used theoretically driven linear models on the same postures [11,21,22]. This includes the orthostatic stroke volume (SV) drop below its clinostatic level, which is accompanied by a compensatory rapid initial (immediate and transient) heart rate (HR) rise, with a quick decline to a level above clinostatic, and a rapid initial (immediate and transient) cardiac output (CO) increase, with a later fast drop below the clinostatic level. It also includes a rapid orthostatic drop in cardiac contractility (as indicated by the rise in pre-ejection period, or PEP), an initial orthostatic vasodilation (indicated by the systemic vascular resistance, or SVR, drop) followed by prolonged vasoconstriction (indicated by the SVR increase), as well as rapid initial drops in systolic and diastolic blood pressure (SBP and DBP) with later restoration (re- or over-shooting). The concordance with earlier studies, along with the between-session congruence of the responses observed in this study (refer to the S Materials.I), confirmed the reliability and robustness of the present findings in the fibromyalgia (FM) group obtained by this non-linear analytical approach. However, probable mechanisms related to the responses and their impairment observed in the FM group cannot be fully or firmly explained with reference to prior studies (see S Materials.II for further discussion).

S Materials.VII. Exploration of various strategies for rebalancing central and peripheral mechanisms to restore body water and blood volume redistribution in response to orthostatic and clinostatic challenges (this section also includes perspectives on the development of new technologies for managing and preventing pain syndromes.

The findings from this study provide an objective physiological basis for clinical recommendations on alternative or complementary medical interventions for pain management. This can include the use of various gadgets, behavioral techniques, and physiotherapeutic procedures for patients with different chronic or acute pain conditions. These interventions have the potential to enhance or stimulate the efficiency of the indicated clino-orthostatic conditioning effect on hemodynamics.

Examples include wearing positive pressure devices such as underbust (lumbar, lumbosacral, sacroiliac) corsets, belts, or back braces made from soft cotton/elastic blends, canvas, flexible rubber materials, or materials with ribbon or of shoelace style [31,32], as well as anti-gravity garments (i.e., simulating reduced gravity by inflatable air bladders over the abdomen, pelvis, thighs, and/or calves) like anti-G suits [33–35] and other lower-body positive pressure devices [36,37]. Additionally, upper-body garments for negative-pressure ventilation, such as cuirasses or jacket ventilators, could also be considered [38,39]. These interventions can provide abdominal/lower-body compression, affecting muscular vessels of the abdomen or calf muscles, or upper-body/thorax decompression. They may be particularly beneficial for patients

experiencing various chronic or acute pain conditions, spanning from menstrual cramps and headaches to back pain, arthritis, and fibromyalgia (FM). This may be especially relevant when these conditions are linked to inadequate venous blood return to the heart for normal blood pressure (BP) regulation by cardiac preload (indicative of increased cardiac output as a physiological resilience mechanism) and heightened reliance on BP regulation through cardiac afterload (indicative of increased systemic vascular resistance (SVR) as a physiological adaptation mechanism), as evidenced in this study. It is recommended that trials supporting these proposals be conducted under the control of the cardiovascular (CV) pain-o-metry procedure.

If properly tailored to an individual's specific anatomy and physiology, lower-body garments can facilitate increased skeletal muscle contractions in the abdomen and/or lower extremities, along with raising intra-abdominal pressure. This promotes the mobilization of unstressed blood volume to augment stressed volume, resulting in rapid venous blood return (the shift of extended blood volume) to the thorax and heart during standing, particularly from the splanchnic and local skeletal muscular vessels, thereby improving cardiac output (CO). Similarly, appropriately fitted upper-body garments can contribute to enhancing the decrease in intrathoracic pressure during inspiration, leading to a more negative right atrial pressure relative to the rest of the body, thus augmenting the gradient for venous blood return to the heart and further improving CO during standing [38]. Additional simple behavioral techniques or physiotherapeutic procedures, such as increasing respiration rate combined with deeper breaths just before and during standing, holding one's breath after a deep inspiration prior to standing (avoiding excessive effort to prevent collapsing the great veins entering the thorax), assuming a passive leg raising position (lying flat on the back with legs elevated approximately 200–300 mm above the heart, distinct from -15° to -30° head-down tilts), and immersing in warm water with the head out, alongside other methods for expanding body water volume, can also enhance the gradient of blood flow for systemic venous return. These approaches facilitate the mobilization of additional blood from the

gastrointestinal circulation and legs, thereby augmenting circulatory volume, cardiac preload, and output [4,40–43]. Additional complementary techniques may involve training the muscles of the thorax, abdomen, and legs through practices such as yoga, physiotherapy, and regular exercise. However, it is crucial to personalize these approaches based on individual CV phenotypes to ensure an effective therapeutic response [44].

As these gadgets, techniques, and procedures enhance the physiological resilience of BP regulation by boosting cardiac preload, they offer a protective mechanism against developing a type of vasoconstriction-induced hypertension with systemic hypoperfusion and extra load on the heart. The findings also suggest that in cases of comorbid pain and hypertension, where a 'BP-related hypoalgesia' phenomenon is observed, antihypertensive medications with diuretic effects (such as thiazide diuretics, mineralocorticoid receptor blockers, calcium channel blockers, and α_1-adrenergic blockers) should be avoided, as they may increase central hypovolemia. Additionally, dietary recommendations with sodium restrictions can have a similar negative impact. Furthermore, medications with vasodilatory effects (including angiotensin-converting enzyme inhibitors, angiotensin II receptor blockers, direct renin inhibitors, β-adrenergic blockers, or centrally acting α-agonists) might disrupt the vasoconstriction 'adaptation to pain' mechanism, potentially worsening pain severity and chronicity unless combined with strategies to restore cardiac preload, such as improving venous blood return, which serves as a physiological resilience mechanism.

An important implication of these findings is the identification of at-risk populations and the advice on appropriate countermeasures. For example, conditions or situations that lead to blood volume contraction (like intense sweating, diuresis, or fluid deposition in the skin and muscles)

with a tendency toward cardiac afterload regulation of BP should be addressed with procedures to increase stressed or effective blood volume, such as enhanced salt and water intake.

S Materials.VIII. Insights into the application of the data-driven analytical technique employed in the study, particularly in relation to the regulatory mechanisms underlying hemodynamic and cardiovascular fluctuations during steady states.

The adaptive model-fitting approach used in this study not only uncovers mechanisms underlying aperiodic changes in hemodynamic and cardiovascular (CV) states, such as initial rapid clino-orthostatic responses and their slower follow-up trends, but also identifies mechanisms involved in the slow and fast periodic fluctuations of these processes during prolonged steady states. This approach could open new avenues for non-invasive studies, offering insights into the cardiorespiratory coordination necessary for efficient fluid management. Specifically, it would elucidate the mechanisms responsible for pumping lymphatic fluid and venous blood from the periphery to the heart, as well as those ensuring effective blood pumping from the heart to the periphery. Additionally, it would allow for an examination of the health status of the organs and systems involved in these processes and facilitate their correction and management.

These processes are crucial for maintaining blood pressure (BP) and volume within the homeostatic range, supporting continuous and smooth blood flow to body tissues. Maintaining arterial BP and volume within this range is contingent on the timely, smooth, and efficient mobilization of stressed and unstressed venous blood volume, as well as lymphatic fluid, back into arterial circulation [45,46]. This is facilitated through various active (intrinsic) and passive (extrinsic) pumping mechanisms, including the lymphatic pump, lung ventilation (via tidal volume and respiration rate), and skeletal muscle activity [46–49]. Fluctuations in these

mechanisms are compensatorily smoothed by variations in cardiac inter-beat intervals. For instance, neural regulation of heart period length dampens oscillations in left ventricular stroke volume (SV), which naturally fluctuate due to breathing mechanics impacting venous return [50,51].

During inspiration, right ventricular SV increases as negative intrathoracic pressure and positive intraabdominal pressure enhance venous return to the thoracic vena cava, while expiration reverses this process. Afferent neural signals originating from the cardiorespiratory mechanoreceptors are considered to inhibit vagal output at the nucleus ambiguous, leading to a shortened heart period during inspiration, which decreases cardiac filling time and, consequently, cardiac preload. Conversely, during expiration, the heart period lengthens, increasing cardiac filling time and thus cardiac preload. This dynamic counteracts the breathing pumping mechanism, which increases venous blood return to the atrium and cardiac preload during inspiration, while decreasing it during expiration. This alternation between bradycardia and tachycardia, known as respiratory sinus arrhythmia (RSA), reflects the modulation of heart rate (HR) by the Bainbridge reflex and occurs within the high-frequency band of heart rate variability (HRV) [52]. The Bainbridge reflex and thus RSA buffers fluctuations in right ventricular SV induced by breathing, as sensed by low-pressure or volume mechanoreceptors, contributing to cardiac effects alongside circulatory and renal influences, thereby smoothing blood flow into the pulmonary circulation [51,53–55]. Thus, as a result, under normal conditions, RSA buffers (reduces) BP fluctuations within the respiration frequency range, known as Traube-Hering waves, thereby stabilizing BP [56]. However, if this damping mechanism is incomplete—due to slower pulses of fluid volume returning from the lymphatic system or disrupted central/peripheral circulation—residual oscillations in left ventricular stroke volume can lead to a combination of fast (Traube-Hering waves) and slow (Mayer waves) fluctuations in arterial pressure [53,56–58]. These oscillations are further regulated by respiratory, cardiac, and vascular

baroreflex feedback control mechanisms, which influence circulatory dynamics at different rates. Some of these effects are reflected in the low-frequency band of HRV [50]. The described mechanisms are analogous to the use of active and passive vibration and pulsation dampers in engineering, where such disturbances threaten the normal functioning of complex dynamic systems during routine but extended operations (e.g., cruising modes) or during short but critical conditions (e.g., afterburner modes). By reducing vibrations and pulsations, dampers—whether in engineered systems like airplane engines or natural systems like the CV system in the human body—help prevent rapid wear and tear, ultimately protecting the entire system from potential damage.

Given the critical role of the venous and lymphatic systems in maintaining blood volume to support arterial pressure within homeostatic ranges, it is proposed that cardiopulmonary baroreceptors primarily detect and respond to reductions in central blood volume during critical hemodynamic conditions, with a secondary role in addressing elevations [48]. This contrasts with the effects of arterial baroreceptors, as both systems work together to regulate HR and SV, adjusting cardiac output (CO) in response to changes in effective circulating volume [59,60]. These reflexes generate signals to coordinate fluctuations between the arterial system—by modulating heart pumping and systemic vascular resistance (SVR)—and the venous and lymphatic systems, by adjusting venous compliance, respiration, and lymphatic pumping activities [61,62]. This coordination ensures the efficient mobilization of venous blood and lymph, helping to restore and maintain arterial blood volume and pressure both at rest and in response to various physiological demands.

For example, lymph flow in the ducts is driven largely by intrinsic lymphatic pumping, with a pacemaker activity rate of approximately 1–10 beats per minute [46,63,64]. In contrast, venous

return primarily relies on extrinsic fluctuations in intrathoracic and intraabdominal pressures, driven by breathing activity at a rate of approximately 12–20 breaths per minute in adults [50,65]. These rates correspond to the low- and high-frequency ranges of HRV, reflecting the effects of cardiac baroreflex and the Bainbridge reflex (RSA) as compensatory mechanisms that smooth blood flow through and out of the heart. During inspiration and cardiac baroreceptor unloading, heart rate increases as interbeat intervals shorten, whereas during expiration and baroreceptor loading, heart rate decreases as interbeat intervals lengthen. This process is further modulated by the respiratory baroreflex, which influences breathing through feedback regulation, while other baroreflexes affecting cardiac contractility and vascular tone [51,62]. In addition to the periodic lymphatic and breathing pumping activities with their feedback mechanisms, slow and very slow rhythmic muscle contractions also play a role in gathering fluid through the lymphatic and venous systems for return to the heart [66]. Together, these mechanisms contribute to the overall complex regulation of blood flow dynamics. The performance of these pumping systems is generally robust against perturbations in fluid dynamics (pressure and flow) under euhydration conditions. However, significant disruptions in fluid balance, such as hypo- or hyper-hydration states, can lead to notable dysfunctions in the operation of these mechanisms [51,60].

For example, the effective degrees of freedom (edf) measures, which represent the actual turning points of hemodynamic and cardiovascular (CV) curves obtained through a data-driven analytical technique, can, when associated with HR fluctuations in the high and low frequency bands, indicate whether respiratory and cardiac baroreflex activities correspond to passive venous flow (driven by extrinsic breathing-related mechanisms that establish venous pressure gradients) or active lymphatic flow (driven by intrinsic pacemaker mechanisms), respectively [46,61]. The present study indicated lower edf values in the high-frequency band of HR fluctuations, associated with respiratory activity, in fibromyalgia (FM) patients. These patients

exhibited approximately 15–16 respiratory cycles per minute, compared to around 18 cycles per minute in healthy participants, across the entire procedure, including both lying and standing postures in both sessions. Although lying and standing may differently affect periodic fluctuations in hemodynamic and CV processes [53,57], this study employed a more conservative analysis, not considering posture as an additional impact factor, thereby reserving a more detailed posture-related analysis for future research. Recent studies highlight that breathing is closely regulated by the respiratory baroreflex, which responds to arterial BP changes by adjusting the breathing rate and depth. This helps maintain BP through the regulation of venous return, acting as a cardiac preload mechanism. Lower systolic BP (SBP) typically increases breathing activity [48,67]. Since SBP levels were similar between the groups, the lower respiratory rate in FM patients suggests that BP in this group was maintained by an alternative mechanism—namely, increased SVR due to a deficit in venous return, supporting the main findings in the study.

The nearly identical fluctuations in heart rate (HR), an indicator of cardiac chronotropy, and pre-ejection period (PEP), an indicator of cardiac contractility (inotropy), in the low-frequency band (approximately 6–7 cycles per minute) between FM patients and healthy participants suggest that a compensatory cardiac baroreflex mechanism functions similarly in both groups. This also suggests that lymphatic pumping, although slower than venous return, functioned properly in both groups. However, this observation only reflects the proper functioning of the intrinsic pacemaker activity of lymphatic pumping and does not provide information on the sufficiency of fluid return volume. Normally, bradycardia increases SV by allowing more time for diastolic filling, which raises end-diastolic volume and stretches cardiac muscle fibers, thus enhancing contraction force in parallel and potentially increasing SV. However, in challenging situations such as hypohydration, as observed in FM patients, a simultaneous decrease in inotropy offsets this adjustment. This is highlighted by the key finding of a reversed close coupling between

chronotropic and inotropic variations in the patient group, particularly during aperiodic orthostatic hemodynamic responses. The reduction in contractility alters the relationship between muscle fiber length and contraction force, leading to either no change or even a reduction in SV despite bradycardia. In fact, the fluid return deficit impacting cardiac preload hemodynamics may be suggested by the difference in SV fluctuations between the FM and healthy groups, with a higher rate in the FM group (around 5 cycles per minute) compared to the healthy group (around 3 cycles per minute). However, this deficit could be more accurately assessed using a different indicator, such as the positive coupling of HRV in the low-frequency band with SV during orthostatic postures (sitting or standing), which present a greater challenge to hemodynamic stability in the FM group due to the added impact of central hypovolemia. Thus, this coupling plays a key role in damping fluctuations in fluid return from the lymphatic system, which become detectable during periods of fluid volume deficits (unpublished findings). This is similar to the positive coupling of HRV within the respiratory frequency band and SV, a key mechanism that dampens respiratory-induced venous return fluctuations. This coupling also becomes more detectable during sitting or standing, as these states challenge hemodynamic stability due to central hypovolemia, in individuals experiencing hypohydration, particularly those prone to hypotension or hypo-volemic subtype of hypertension [51,53,60].

All the aforementioned regulations of cardiac preload processes affected cardiac output (CO) fluctuations, which occurred at approximately half the rate in the FM group (about 3–4 cycles per minute) compared to the healthy group (around 7 cycles per minute). These fluctuations corresponded to SVR fluctuations in the groups, with rates of about 4.0 cycles per minute in the FM group and approximately 7.0 cycles per minute in the healthy group. This correspondence extends to SBP fluctuations, with approximately 3 cycles per minute in the FM group and 6–7 cycles per minute in the healthy group. In the end, diastolic BP exhibited a lesser impact from the primary preload fluctuations, maintaining the previous ratio's consistency, with fluctuations

of approximately 2 cycles per minute in the FM group and 4 cycles per minute in the healthy group. All these findings incorporate periodic fluctuations in hemodynamic and CV processes as additional components of the pain-o-metric profile outlined by the main findings. The differences in fluctuation ranges between the groups, observed in CO, SVR, SBP, and DBP, may be attributed to the varying influence of CO and SVR on BP regulation. In FM patients, a higher SVR reduces vascular compliance (i.e., increases functional arterial stiffness), impairing the vessels' ability to dampen pressure fluctuations in this low frequency band. Normally, compliant arteries expand after each heartbeat to accommodate surges in blood flow, followed by recoil, which helps maintain continuous, steady, and smooth blood flow (Windkessel model). In this case, these blood pressure oscillations may have protective effects on end organs, such as the kidneys [58,68,69]. In FM patients, this damping effect is diminished, leading to reduced vascular fluctuations and decreased blood flow. In contrast, healthy participants with more compliant arteries show more effective expansion and greater fluctuations in SVR, SBP, and DBP, as illustrated in the respective figures. Previous studies have shown that these fluctuations are controlled by carotid baroreceptors, which continuously inhibit vascular tone, thereby enhancing vascular flexibility in response to surges in blood flow. This was confirmed by surgical severing of the afferent neural fibers from these receptors, which resulted in a reduction of BP fluctuations in the low-frequency band, near 0.1 Hz [70]. Thus, while fluctuations in cardiac preload are primarily regulated by active, reflexive cardiac damping mechanisms, fluctuations in cardiac afterload are mainly moderated by passive vascular damping mechanisms, such as arterial elasticity, with secondary influences from respiratory, cardiac, and vascular baroreflex feedback control mechanisms (as discussed above). These additional findings suggest that the edf value in the dynamics of certain CV measures could serve as an alternative indicator of reduced functional compliance of resistance vessels (increased functional arterial stiffness) in response to central hypovolemia and total hypohydration. This condition, characterized by low magnitude and infrequent blood flow surges (< 0.1 Hz) requiring damping, could help identify

individuals suffering from or at risk of chronic pain, complementing or replacing those identified through the pain-o-metric pattern observed in the main findings.

S Materials.IX. The electrode placements for electrocardiogram (ECG) and impedance cardiogram (ICG) acquisitions adhered to the study's established protocols for hemodynamic and cardiovascular measurements.

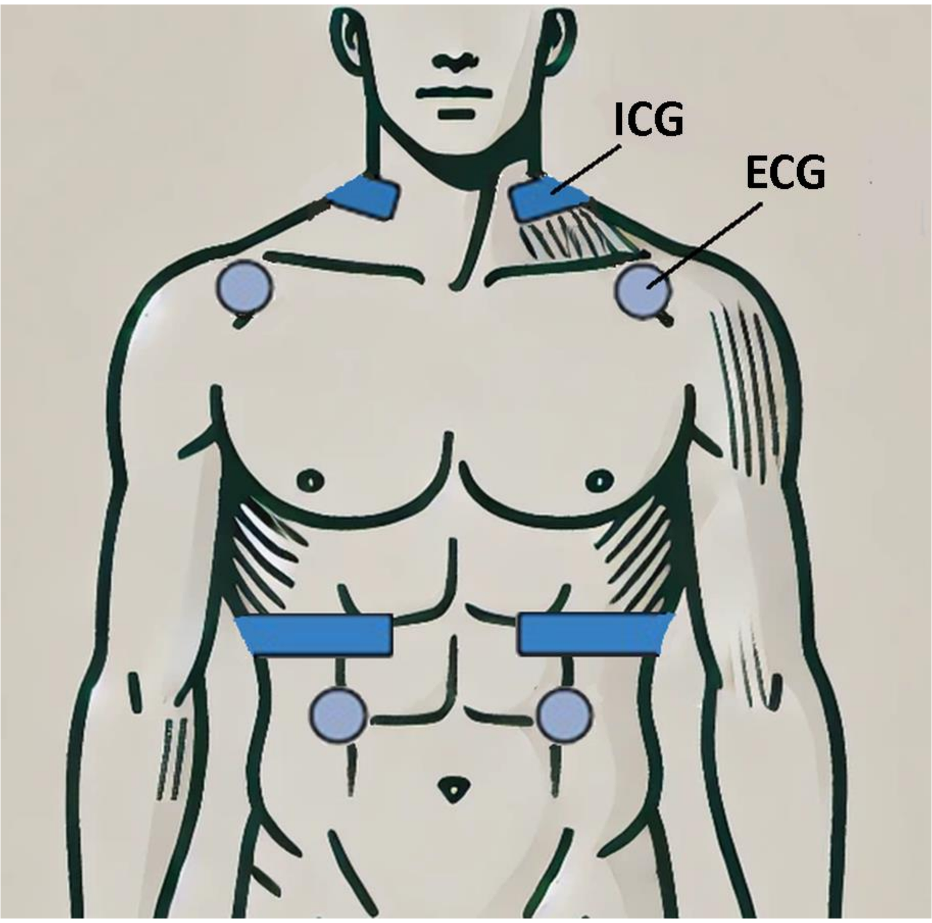

S8 Fig. Schema of the electrode placements for electrocardiogram (ECG; in light blue) and impedance cardiogram (ICG; in dark blue) based on the specifications provided by the Task Force Monitor vendor (CNSystems, Graz, Austria). For further details, please refer to the methods section of the article.

References

1. Berry NM, Rickards CA, Newman DG. Squat-stand test response following 10 consecutive episodes of head-up tilt. Aviat Sp Environ Med. 2006;77: 1125–1130.

2. Berry NM, Rickards CA, Newman DG. Acute cardiovascular adaptation to 10 consecutive episodes of head-up tilt. Aviat Sp Environ Med. 2006;77: 494–499.

3. Harms MPM, van Lieshout JJ. Cerebrovascular and cardiovascular responses associated with orthostatic intolerance and tachycardia. Clin Auton Res. 2001;11: 35–38. doi:10.1007/BF02317800

4. Davydov DM. Linking the heart and pain: physiological and psychophysiological mechanisms. 1st ed. In: Rajendram R, Patel VB, Preedy VR, Martin CR, editors. Features and Assessments of Pain, Anaesthesia, and Analgesia (a volume of The Neuroscience of Pain, Anesthetics, and Analgesics). 1st ed. London, UK: Academic Press Inc.; 2022. pp. 211–223. doi:10.1016/B978-0-12-818988-7.00011-X

5. Davydov DM. Health in medicine: The lost graal. J Psychosom Res. 2018;111: 22–26. doi:10.1016/j.jpsychores.2018.05.006

6. Sheikh NA, Phillips AA, Ranada S, Lloyd M, Kogut K, Bourne KM, et al. Mitigating Initial Orthostatic Hypotension: Mechanistic Roles of Muscle Contraction Versus Sympathetic Activation. Hypertension. 2022;79: 638–647. doi:10.1161/HYPERTENSIONAHA.121.18580

7. Blomqvist CG, Stone HL. Cardiovascular Adjustments to Gravitational Stress. Compr Physiol (handb Physiol Cardiovasc Syst Peripher Circ Organ Blood Flow Chapter 28, 1983). 2011; 1025–1063. doi:10.1002/cphy.cp020328

8. Patel K, Rössler A, Lackner HK, Trozic I, Laing C, Lorr D, et al. Effect of postural changes on cardiovascular parameters across gender. Med (United States). 2016;95:

e4149. doi:10.1097/MD.0000000000004149

9. Jordan J, Biaggioni I, Grassi G, Fedorowski A, Kario K. When Blood Pressure Increases with Standing: Consensus Definition for Diagnosing Orthostatic Hypertension. Blood Press. 2023;32: 2161871. doi:10.1080/08037051.2022.2161871

10. Klijn E, Niehof S, Johan Groeneveld AB, Lima AP, Bakker J, van Bommel J. Postural change in volunteers: sympathetic tone determines microvascular response to cardiac preload and output increases. Clin Auton Res. 2015;25: 347–354. doi:10.1007/s10286-015-0286-x

11. Stewart JM, Clarke D. “He’s dizzy when he stands up”: An introduction to initial orthostatic hypotension. J Pediatr. 2011;158: 499–504. doi:10.1016/j.jpeds.2010.09.004

12. Harms MPM, Finucane C, Pérez-Denia L, Jurachek S, van Wijnen VK, Lipsitz LA, et al. Systemic and cerebral circulatory adjustment within the first 60 s after active standing: An integrative physiological view. Auton Neurosci Basic Clin. 2021;231: 102756. doi:10.1016/j.autneu.2020.102756

13. Selvarajah V, Connolly K, McEniery C, Wilkinson I. Skin Sodium and Hypertension: a Paradigm Shift? Curr Hypertens Rep. 2018;20. doi:10.1007/s11906-018-0892-9

14. Wiig H, Luft FC, Titze JM. The interstitium conducts extrarenal storage of sodium and represents a third compartment essential for extracellular volume and blood pressure homeostasis. Acta Physiol. 2018;222. doi:10.1111/apha.13006

15. Hall JE, Granger JP, do Carmo JM, da Silva AA, Dubinion J, George E, et al. Hypertension: Physiology and pathophysiology. Compr Physiol. 2012;2: 2393–2442. doi:10.1002/cphy.c110058

16. Dempsey JA. New perspectives concerning feedback influences on cardiorespiratory control during rhythmic exercise and on exercise performance. J Physiol. 2012;590: 4129–

44. doi:10.1113/jphysiol.2012.233908

17. Moore JP, Simpson LL, Drinkhill MJ. Differential contributions of cardiac, coronary and pulmonary artery vagal mechanoreceptors to reflex control of the circulation. J Physiol. 2022;600: 4069–4087. doi:10.1113/JP282305

18. Halliwill JR. Virtual conductance, real hypotension: What happens when we stand up too fast? J Appl Physiol. 2007;103: 421–422. doi:10.1152/japplphysiol.00544.2007

19. Casey DP, Hart EC. Cardiovascular function in humans during exercise: Role of the muscle pump. J Physiol. 2008;586: 5045–5046. doi:10.1113/jphysiol.2008.162123

20. González-Alonso J, Mortensen SP, Jeppesen TD, Ali L, Barker H, Damsgaard R, et al. Haemodynamic responses to exercise, ATP infusion and thigh compression in humans: Insight into the role of muscle mechanisms on cardiovascular function. J Physiol. 2008;586: 2405–2417. doi:10.1113/jphysiol.2008.152058

21. Tanaka H, Sjöberg BJ, Thulesius O. Cardiac output and blood pressure during active and passive standing. Clin Physiol. 1996;16: 157–170. doi:10.1111/j.1475-097X.1996.tb00565.x

22. Thomas KN, Cotter JD, Galvin SD, Williams MJA, Willie CK, Ainslie PN. Initial orthostatic hypotension is unrelated to orthostatic tolerance in healthy young subjects. J Appl Physiol. 2009;107: 506–517. doi:10.1152/japplphysiol.91650.2008

23. Tschakovsky ME, Matusiak K, Vipond C, McVicar L. Lower limb-localized vascular phenomena explain initial orthostatic hypotension upon standing from squat. Am J Physiol Circ Physiol. 2011;301: H2102–H2112. doi:10.1152/ajpheart.00571.2011

24. Sheriff DD, Nådland IH, Toska K. Hemodynamic consequences of rapid changes in posture in humans. J Appl Physiol. 2007;103: 452–458. doi:10.1152/japplphysiol.01190.2006

25. van Wijnen VK, Finucane C, Harms MPM, Nolan H, Freeman RL, Westerhof BE, et al. Noninvasive beat-to-beat finger arterial pressure monitoring during orthostasis: a comprehensive review of normal and abnormal responses at different ages. J Intern Med. 2017;282: 468–483. doi:10.1111/joim.12636

26. Gliemann L, Vestergaard Hansen C, Rytter N, Hellsten Y. Regulation of skeletal muscle blood flow during exercise. Current Opinion in Physiology. Elsevier Ltd; 2019. pp. 146–155. doi:10.1016/j.cophys.2019.05.001

27. Hall ME, Hall JE. Pathogenesis of Hypertension. 3rd ed. In: Bakris GL, Sorrentino MJ, editors. Hypertension: A Companion to Braunwald's Heart Disease. 3rd ed. Philadelphia, PA, USA: Elsevier; 2018. pp. 33–51.

28. Rakova N, Kitada K, Lerchl K, Dahlmann A, Birukov A, Daub S, et al. Increased salt consumption induces body water conservation and decreases fluid intake. J Clin Invest. 2017;127: 1932–1943. doi:10.1172/JCI88530

29. Drinkhill MJ, Mary DA. The effect of stimulation of the atrial receptors on plasma cortisol level in the dog. J Physiol. 1989;413: 299–313. doi:10.1113/jphysiol.1989.sp017655

30. Zaidi A, Benitez D, Gaydecki PA, Vohra A, Fitzpatrick AP. Haemodynamic effects of increasing angle of head up tilt. Heart. 2000;83: 181–184. doi:10.1136/heart.83.2.181

31. Pope MH, Phillips RB, Haugh LD, Hsieh CYJ, Macdonald L, Haldeman S. A prospective randomized three-week trial of spinal manipulation, transcutaneous muscle stimulation, massage and corset in the treatment of subacute low back pain. Spine (Phila Pa 1976). 1994;19: 2571–2577. doi:10.1097/00007632-199411001-00013

32. Sato N, Sekiguchi M, Kikuchi S, Shishido H, Sato K, Konno S. Effects of long-term corset wearing on chronic low back pain. Fukushima J Med Sci. 2012;58: 60–65.

doi:10.5387/fms.58.60

33. Fox JL. Orthostatic hypotension following bilateral percutaneous cordotomy. Acta Neurochir (Wien). 1971;24: 219–224. doi:10.1007/bf01400558

34. Seaworth JF, Jennings TJ, Howell LL, Frazier JW, Goodyear CD, Grassman ED. Hemodynamic effects of anti-G suit inflation in a 1-G environment. J Appl Physiol. 1985;59: 1145–1151. doi:10.1152/jappl.1985.59.4.1145

35. Montmerle S, Linnarsson D. Cardiovascular effects of anti-G suit inflation at 1 and 2 G. Eur J Appl Physiol. 2005;94: 235–241. doi:10.1007/s00421-005-1331-6

36. Takacs J, Anderson JE, Leiter JRS, MacDonald PB, Peeler JD. Lower body positive pressure: An emerging technology in the battle against knee osteoarthritis? Clin Interv Aging. 2013;8: 983–991.

37. Stucky F, Vesin JM, Kayser B, Uva B. The effect of lower-body positive pressure on the cardiorespiratory response at rest and during submaximal running exercise. Front Physiol. 2018;9. doi:10.3389/fphys.2018.00034

38. Corrado A, Gorini M. Long-term negative pressure ventilation. Respir Care Clin N Am. 2002;8: 545–557. doi:10.1016/s1078-5337(02)00026-6

39. Thomson A. The role of negative pressure ventilation. Arch Dis Child. 1997;77: 454–458. doi:10.1136/adc.77.5.454

40. Berlin DA, Bakker J. Understanding venous return. Intensive Care Med. 2014;40: 1564–1566. doi:10.1007/s00134-014-3379-4

41. Pendergast DR, Lundgren CEG. The underwater environment: Cardiopulmonary, thermal, and energetic demands. J Appl Physiol. 2009;106: 276–283. doi:10.1152/japplphysiol.90984.2008

42. Hoffmann U, Dräger T, Steegmanns A, Koesterer T, Linnarsson D. Influence of combined exercise and gravity transients and apnea on hemodynamics. Eur J Appl Physiol. 2009;106: 589–597. doi:10.1007/s00421-009-1052-3

43. Boulain T, Achard JM, Teboul JL, Richard C, Perrotin D, Ginies G. Changes in BP induced by passive leg raising predict response to fluid loading in critically ill patients. Chest. 2002;121: 1245–1252. doi:10.1378/chest.121.4.1245

44. Davydov DM, Shahabi L, Naliboff B. Cardiovascular phenotyping for personalized lifestyle treatments of chronic abdominal pain in Irritable Bowel Syndrome: A randomized pilot study. Neurogastroenterol Motil. 2019;31: e13710. doi:10.1111/nmo.13710

45. Margaris KN, Black RA. Modelling the lymphatic system: Challenges and opportunities. J R Soc Interface. 2012;9: 601–612. doi:10.1098/rsif.2011.0751

46. Zawieja DC. Contractile physiology of lymphatics. Lymphat Res Biol. 2009;7: 87–96. doi:10.1089/lrb.2009.0007

47. Stewart JM, Rivera E, Clarke DA, Baugham IL, Ocon AJ, Taneja I, et al. Ventilatory baroreflex sensitivity in humans is not modulated by chemoreflex activation. Am J Physiol - Hear Circ Physiol. 2011;300: H1492–H1500. doi:10.1152/ajpheart.01217.2010

48. McMullan S, Pilowsky PM. The effects of baroreceptor stimulation on central respiratory drive: A review. Respir Physiol Neurobiol. 2010;174: 37–42. doi:10.1016/j.resp.2010.07.009

49. Baekey DM, Molkov YI, Paton JFR, Rybak IA, Dick TE. Effect of baroreceptor stimulation on the respiratory pattern: Insights into respiratory-sympathetic interactions. Respir Physiol Neurobiol. 2010;174: 135–145. doi:10.1016/j.resp.2010.09.006

50. McMullen MK, Whitehouse JM, Shine G, Towell A. Respiratory and non-respiratory

sinus arrhythmia: Implications for heart rate variability. J Clin Monit Comput. 2012;26: 21–28. doi:10.1007/s10877-011-9327-8

51. Reyes del Paso GA, Montoro CI, Davydov DM, Duschek S. The cardiac, vasomotor and myocardial branches of the baroreflex in hypotension: Indications of reduced venous return to the heart. Clin Auton Res. 2024.

52. Taha BH, Simon PM, Dempsey JA, Skatrud JB, Iber C. Respiratory sinus arrhythmia in humans: An obligatory role for vagal feedback from the lungs. J Appl Physiol. 1995;78: 638–645. doi:10.1152/jappl.1995.78.2.638

53. Kotani K, Takamasu K, Jimbo Y, Yamamoto Y. Postural-induced phase shift of respiratory sinus arrhythmia and blood pressure variations: Insight from respiratory-phase domain analysis. Am J Physiol - Hear Circ Physiol. 2008;294. doi:10.1152/ajpheart.00680.2007

54. Elstad M, Toska K, Chon KH, Raeder EA, Cohen RJ. Respiratory sinus arrhythmia: Opposite effects on systolic and mean arterial pressure in supine humans. J Physiol. 2001;536: 251–259. doi:10.1111/j.1469-7793.2001.t01-1-00251.x

55. Elstad M, Walløe L, Holme NLA, Maes E, Thoresen M. Respiratory sinus arrhythmia stabilizes mean arterial blood pressure at high-frequency interval in healthy humans. Eur J Appl Physiol. 2015;115: 521–530. doi:10.1007/s00421-014-3042-3

56. Barnett WH, Latash EM, Capps RA, Dick TE, Wehrwein EA, Molkov YI. Traube–Hering waves are formed by interaction of respiratory sinus arrhythmia and pulse pressure modulation in healthy men. J Appl Physiol. 2020;129: 1193–1202. doi:10.1152/japplphysiol.00452.2020

57. Grabov E, Sullivan P, Wang S, Goldstein DS. Tilt-evoked, breathing-driven blood pressure oscillations: Independence from baroreflex-sympathoneural function. Clin Auton

Res. 2024;34: 125–135. doi:10.1007/s10286-024-01022-7

58. Julien C. The enigma of Mayer waves: Facts and models. Cardiovasc Res. 2006;70: 12–21. doi:10.1016/j.cardiores.2005.11.008

59. Davydov DM, Naliboff B, Shahabi L, Shapiro D. Asymmetries in reciprocal baroreflex mechanisms and chronic pain severity: Focusing on irritable bowel syndrome. Neurogastroenterol Motil. 2018;30: e13186. doi:10.1111/nmo.13186

60. Davydov DM, de la Coba P, Contreras-Merino AM, Reyes del Paso GA. Impact of homeostatic body hydration status, evaluated by hemodynamic measures, on different pain sensitization paths to a chronic pain syndrome. Sci Rep. 2024;14: 1908. doi:10.1038/s41598-024-52419-3

61. Zena LA, Da Silva GSF, Gargaglioni LH, Bícego KC. Baroreflex regulation affects ventilation in the cururu toad Rhinella schneideri. J Exp Biol. 2016;219: 3605–3615. doi:10.1242/jeb.144774

62. Segers LS, Nuding SC, Ott MM, O'Connor R, Morris KF, Lindsey BG. Blood pressure drives multispectral tuning of inspiration via a linked-loop neural network. J Neurophysiol. 2020;124: 1676–1697. doi:10.1152/jn.00442.2020

63. McHale NG, Meharg MK. Co-ordination of pumping in isolated bovine lymphatic vessels. J Physiol. 1992;450: 503–512. doi:10.1113/jphysiol.1992.sp019139

64. Moore JE, Bertram CD. Lymphatic System Flows. Annu Rev Fluid Mech. 2018;50: 459–482. doi:10.1146/annurev-fluid-122316-045259

65. Davydov DM, Naliboff B, Shahabi L, Shapiro D. Baroreflex mechanisms in Irritable Bowel Syndrome: Part I. Traditional indices. Physiol Behav. 2016;157: 102–108. doi:10.1016/j.physbeh.2016.01.042

66. Davydov DM, Boev A. Heart disease risk assessment (patent US10463260B1). USA:

United States Patent and Trademark office; World Intellectual Property Organization (International Bureau); US10463260B1; WO2020227278A1, 2019.

67. Brunner MJ, Sussman MS, Greene AS, Kallman CH, Shoukas AA. Carotid sinus baroreceptor reflex control of respiration. Circ Res. 1982;51: 624–636. doi:10.1161/01.RES.51.5.624

68. Nafz B, Stegemann J, Bestie MH, Richter N, Seeliger E, Schimke I, et al. Antihypertensive effect of 0.1-Hz blood pressure oscillations to the kidney. Circulation. 2000;101: 553–557. doi:10.1161/01.CIR.101.5.553

69. Nafz B, Wagner CD, Persson PB. Endogenous nitric oxide buffers blood pressure variability between 0.2 and 0.6 Hz in the conscious rat. Am J Physiol - Hear Circ Physiol. 1997;272. doi:10.1152/ajpheart.1997.272.2.h632

70. Di Rienzo M, Parati G, Radaelli A, Castiglioni P. Baroreflex contribution to blood pressure and heart rate oscillations: time scales, time-variant characteristics and nonlinearities. Philos Trans A Math Phys Eng Sci. 2009;367: 1301–18. doi:10.1098/rsta.2008.0274
