# Supplementary material for: Hemodynamics in chronic pain: A pathway to multi-modal health risks
Source: PLoS One. 2024 Dec 10;19(12):e0315341. doi: 10.1371/journal.pone.0315341 (PMC11630596; doi:10.1371/journal.pone.0315341)
Supplement: Attachment — Submitted filename: Response to Reviewers.docx [file pone.0315341.s002.docx]

**Replies to comments**

We sincerely appreciate the reviewers for their thoughtful comments and the editor for giving us the opportunity to resubmit the manuscript after revision. We have carefully addressed all the feedback and suggestions, making the necessary revisions to the manuscript. Below, we provide a point-by-point response to the editor's and reviewers' comments.

Editor’s comments:

1. *Please ensure that your manuscript meets* *PLOS ONE's style requirements, including those for file naming.*

**Reply:** Rechecked and corrected according to the PLOS ONE's style requirements.

2. *Please note that PLOS ONE has specific guidelines on code sharing for submissions in which author-generated code underpins the findings in the manuscript. In these cases, we expect all author-generated code to be made available without restrictions upon publication of the work. Please review our guidelines at https://journals.plos.org/plosone/s/materials-and-software-sharing#loc-sharing-code and ensure that your code is shared in a way that follows best practice and facilitates reproducibility and reuse.*

**Reply:** No author-generated code was used in this study. All programs and procedures employed are publicly available, as detailed in the methods section.

3. *Thank you for stating the following financial disclosure: “Gustavo A. Reyes del Paso, the Spanish Ministry of Science and Innovation, co-financed by European Regional Development Fund [PID2022-139731OB-I00]”. Please state what role the funders took in the study. If the funders had no role, please state:* *"**The funders had no role in study design, data collection and analysis, decision to publish, or preparation of the manuscript." If this statement is not correct you must amend it as needed. Please include this amended* *Role of Funder statement in your cover letter; we will change the online submission form on your behalf.*

**Reply:** The Role of the Funder statement has been included in both the cover letter and the corresponding section of the manuscript as follows: “The funders had no role in study design, data collection and analysis, decision to publish, or preparation of the manuscript.”

4. *In this instance it seems there may be acceptable restrictions in place that prevent the public sharing of your minimal data. However, in line with our goal of ensuring long-term data availability to all interested researchers, PLOS’ Data Policy states that authors cannot be the sole named individuals responsible for ensuring data access (*[*http://journals.plos.org/plosone/s/data-availability#loc-acceptable-data-sharing-methods*](http://journals.plos.org/plosone/s/data-availability#loc-acceptable-data-sharing-methods)*). Data requests to a non-author institutional point of contact, such as a data access or ethics committee, helps guarantee long term stability and availability of data. Providing interested researchers with a durable point of contact ensures data will be accessible even if an author changes email addresses, institutions, or becomes unavailable to answer requests. Before we proceed with your manuscript, please also provide non-author contact information (phone/email/hyperlink) for a data access committee, ethics committee, or other institutional body to which data requests may be sent. If no institutional body is available to respond to requests for your minimal data, please consider if there any institutional representatives who did not collaborate in the study, and are not listed as authors on the manuscript, who would be able to hold the data and respond to external requests for data access? If so, please provide their contact information (i.e., email address). Please also provide details on how you will ensure persistent or long-term data storage and availability.*

**Reply:** The requested contact information has been included in the manuscript under the data availability statement.

“Data requests may also be directed to the General Coordinator and Secretary of the Ethics Committee, referencing #: OCT.18/1.PRY and the title "Central Sensitization in Chronic Pain: The Case of Fibromyalgia": <https://www.ujaen.es/gobierno/vicinv/comision-de-etica>”

**Replies to reviewers’ comments**

Reviewer #1:

1 *In the introduction, the authors claim that linear statistical methods were a limitation for analyzing the hemodynamic and CV reactions. As the methodological novelty of this paper originates from implementing non-linear models, it would be beneficial to expand on the current contributions of linear statistical models and where these have resulted in limited or erroneous understanding of hemodynamic processes.*

**Reply:**  Thank you for your comment. We have expanded this section accordingly (p. 5, lines 7-26, and p. 6, lines 1-7).

2 *The statistical test performed and reported table 1 is not mentioned in the text. Please add a description of what has been performed and reported.*

**Reply:**  Thanks. Added.

3 *The results of the administered questionnaires are presented in table 1, yet the description of these tests is described later in the manuscript. I suggest the authors consider reorganizing the flow.*

**Reply:**  The corresponding paragraphs were reorganized accordingly.

4 *In the calculation of the systemic vascular resistance, how did the authors measure or estimate the central venous pressure?*

**Reply:**  We have addressed the issue on page 13, lines 5–7, with references supporting the point.

5 *The paper would benefit from a more detailed description of the statistical models presented on page 17. In the equation, clearly define and reference all variables used within.*

**Reply:**  This point is now addressed in more details on pages 19-20, with references to other paragraphs of the section (pages 15 and 16) where these models and the variables included in the equation are discussed and further clarified.

6 *The results section presents what appear to be the results of F-tests. This procedure should be described in the method’s section.*

**Reply:**  This point is now addressed on page 19 (lines 11-12).

7 *The results section report the fluctuation of the hemodynamic and CV measures as measured with the “edf” values. The significance of this measurement was not clearly apparent from the discussion section, as it appeared that mainly steady-state values and transitions were of interest. What conclusions are drawn from the “edf” values? Also, hemodynamic and CV measures are known to fluctuate with the breathing, could this have played a role in the observed fluctuations?*

**Reply:**  Thank you for your interest in the indicator. Due to the manuscript's length, we initially limited our discussion of this point to a brief remark (see page 52, lines 1-21 of the revised version). However, we have now expanded the discussion to emphasize the additional value of this measure, providing its short analysis at the end of the subsections discussing findings related to each hemodynamic and cardiovascular variable, with further details now included in S Materials.VIII.

8 *The statement “Previous research suggests that these lifestyle factors do not significantly alter the fundamental relationship between mechanisms governing CV processes and pain mechanisms.” requires citations.*

**Reply:**  Thank you. In the cited version of the manuscript, these references were originally placed after the following sentence, which further elaborates on the issue. We have now moved them to the indicated sentence (page 54, line 6).

9 *In the figures, the dashed vertical lines, which are used to delineate periods in that mark a significant between group differences, are hard to follows in some instances. The reviewer suggests modifying the graphical method used to highlight this difference.*

**Reply:** Thank you for your comment. The original high-resolution figures included additional markers indicating areas of significant differences, which were unfortunately not visible in the low-resolution versions. We apologize for this oversight. We have now uploaded the high-resolution figures, where the time windows showing significant between-group differences (FM vs. CT) are marked with horizontal bold-line projections on the Time (X) axis. These are bounded by vertical dashed lines, corresponding to the relevant points along the CV difference curves. The figure legends have also been updated to reflect this clarifying information.

10 *In the Supplemental Materials, the authors show figures comparing the Session 1 and Session 2 hemodynamic and CV measures. Certain measures, such as CO and SVR, show noticeable differences in overall magnitude, shapes, and effective degrees of freedom. Could the authors further comment on this.*

**Reply:** Thank you for your interest in the findings. The section S Materials.I has been enhanced with additional discussions on within-subject effects on hemodynamics across sessions (pages 3-5).

Reviewer #2:

1. *Abstract: a couple of minor suggestions: "sec-by-sec" might be replaced by "second-by-second" (avoiding abbreviations in an abstract); "the chronic disease" might be replaced by "chronic disease" (no article); "enabled the identification" might be replaced by "enables..." (in order to be consistent with the present tense, and to respect that the methodology you are proposing is applicable beyond your work).*

**Reply:** Thank you! The references in the abstract have been corrected.

2. *Intro, first paragraph, page 3: "World" should not be capitalized.*

**Reply:** The word has been corrected.

3. *Table 1: there is no explanation, neither in the caption, nor in the text, of "t-\chi" or "p-value" before the table appears. Someone working in data-driven statistical approaches can guess easily, but for completeness, the former, at least, should be explained somewhere in text or caption.*

**Reply:** Thank you! The necessary explanation has been included in the caption and added to the methods section (page 20, lines 13-16).

4. *Page 9: the title "Procedure, and clinical and psychological testing instruments" is a bit awkward; consider rephrasing to remove the second "and" (maybe: "Procedure and clinical/psychological testing instruments)*

**Reply:** Thank you for the suggestion! The title has been corrected.

5. *Page 12, bottom of the page: "respective data-driven adaptive techniques were used to take into account..." When someone reads this, they expect a list of the techniques, or more description. if you are referring to the rest of the "statistical metrics" section, then you should mention here ("**as detailed in what follows")*

**Reply:** Thank you for the suggestion! The sentence has been corrected.

6. *Page 13, "hence, this non-linear methodology aimed to overcome..." It is not clear that your methodology is non-linear at this point. Also, the definition of "non-linear" versus "linear" is not mathematical here (I don't think), and so some phrases must be included in order to explain what exactly you mean by non-linear vs. linear. is non-linear just regressions that are co-variate adjusted?*

**Reply:** Thank you for highlighting this issue. The indicated sentence, along with the entire paragraph, was indeed misplaced and has now been relocated after the following paragraph, combining them into one (page 14, lines 21-25, and page 15, lines 1-17) to improve the clarity of the discourse.

7. *Page 13, "because of this, a special non-parametric approximation technique..." What is the technique? Why is it special? Can you add some references? How do they accommodate non-linear effects?*

**Reply:** We have included (page 14, lines 20-22) the reference to the package implementing the technique used, along with a section below that elaborates on the details of the technique.

8. *Page 15, "fixed in 240" should be "fixed to 240"*

**Reply:** Thanks. Corrected.

9. *On page 15, you italicize (correctly) "p" for p-value. elsewhere in the manuscript, it is no longer italisized (e.g. bottom of page 17). please correct throughout and make it consistent.*

**Reply:** All relevant designations have been italicized throughout the manuscript.

10. *a number of metrics are used without citation...BIC, AIC. similarly, for bootstrapping discussion, please provide references and/or a description of what it exactly is for those who are less informed. in fact, a lot of the statistical methods section does not include references to the methods you are using. I know some are quite standard, but it would be useful for a reader to quickly refer to a paper for a description of the analysis metric you are using (i.e., to see the formulas, etc.)*

**Reply:** We added more references to literature about the primary mathematical and statistical methods to read along with references to secondary methods.

11. *"Supplementary materials" is sometimes lower case on the second word, sometimes upper case. Please correct throughout.*

**Reply:** Thank you! Corrected.

12. *It is unusual for someone to call Supplementary Materials Davydov (i.e., using their last name in the title). it's confusing. Please just use "Supplementary Materials" throughout the manuscript and in the supplement itself (i.e., remove your name from the section calls).*

**Reply:** Corrected.

13. *Page 17" "non-standardized (B) regression coefficients" ... shouldn't this "B" be lowercase to comply with convention?*

**Reply:** Corrected.

14. *in line with comment 9, perhaps consider italicizing (as is common) all the statistical variables (e.g., those that start on Page 18 results...b,t,p, etc.). it will make it easier to see these values embedded in such dense text.*

**Reply:** Corrected.

15. *Figures 1-7: since in a number of subfigures (particularly (B) and (C)) have the exact same axes, a title might be useful to help people see right away that one plot is unadjusted and the other is adjusted. in fact, you may benefit from putting titles on all the figures (i.e., "Before adjustment of BMI", "After adjustment of BMI", etc.) In some cases it's (a) and (b), i.e., Figure 2. I think the specific co-variate considered (which varies between your figures/metrics) should be included in the figure titles.*

**Reply:** Added to the respective figures to indicate their relevance to analyses before or after adjustments, if permitted by editorial policy, but without specifying particular covariates, as this information is already provided in the figure captions.

16. *Page 21: "with high, low, and very low HR frequency fluctuations" ...these are only two figures (2A) and (2B) corresponding to high and low fluctuations. please correct.*

**Reply:** Thank you! Corrected.

17. *Figure 2, caption: I think there is a typo. in the last line, it should be "fast (C) and slow (D)" and not "fast (B) and slow (C)".*

**Reply:** Thank you! Yes, you are right! Corrected.

18. *All figures: the grey dotted lines are not mentioned in any of the captions (as to what they demarcate). in the text they are not mentioned either, but we can easily infer that it's the intervals you are referring to. please mention the dotted lines so people know what they mean more easily.*

**Reply:** Thank you! The clarification for the vertical dashed lines has been added to the corresponding figures.

19. *following 18), what do the dotted lines correspond to in Figure 2D? for other figures, you at least mention some of the intervals, or that you are showing locations of max deviation. but for 3D, I don't understand at all what the dotted lines are supposed to represent in the curves...*

**Reply:** We have added further clarifications for the vertical dashed lines and the regions they indicate, along with the horizontal bold lines on the X-axis that were not visible in the low-resolution figures. We apologize for the inconsistency.

20. *page 23, "very short random periods of significant differences": please revise. how do you define "significant difference" here, and "random periods"? i don't quite see that in the figure (the overall trend shows differences are high throughout...)*

**Reply:** The sentence has been revised (page 25, lines 8-10).

21. *page 29, "after a common rapid SVR drops" should be "after a common rapid SVR drop" (singular)*

**Reply:** Thank you! Corrected (page 31, line 10).

22. *figures 6 and 7: these appear to be the only two metrics (DBP and SBP) where, after co-variate adjustment, the differences between FM and CT become smaller. can you discuss/explain?*

**Reply:** Thank you for the comment. A paragraph has been added to explain the issue (page 46, lines 4-14):

“In contrast to other hemodynamic and cardiovascular measures, the differences in SBP and DBP activity between the FM and healthy groups during the clino-orthostatic challenge diminished after adjusting for BMI as a covariate (Figs 6D and 7D). This is likely because BMI, a key covariate for these metrics in both the GAMM and GAM models, differed significantly between the groups (Table 1). As a probable mediating factor in the causal mechanisms linking chronic pain and blood pressure (Davydov, 2022), BMI likely contributed to these between-group differences. In contrast, covariates such as age and height, though more relevant to other measures, had a lower and statistically insignificant impact on between-group differences, despite their significant linear effects in the models. These covariates primarily influenced overall between-subject variability, contributing less to between-group differences.”

Reviewer #3:

1. *Many sentences specially in Introduction are overly long, which detracts from their clarity. I suggest revising these sentences to focus on one idea at a time. I suggest breaking up these sentences and focusing on presenting one idea at a time.*

**Reply:** While this suggestion could be valid, it would be more helpful if the reviewer pointed out the specific areas, he found problematic. As the authors and colleagues familiar with the manuscript, we may overlook such sections after having read it multiple times. Nevertheless, we reviewed the text, particularly the introduction, with this in mind and revised some parts to simplify the content (e.g., page 3, line 7; page 4, line 19; page 16, line 17; page 18, line 2; page 24, line 25; page 39, lines 5 and 7).

2. *Please be consistent for the word “nonlinear” or “non-linear”. Choose one for the entire manuscript.*

**Reply:** Corrected.

3. *While the exclusion criteria are comprehensive, it would be helpful to provide more information on the specific FM-related medications allowed in the study. It would be important to clarify how the authors managed potential effects. How were their potential effects on cardiovascular outcomes accounted for?*

**Reply:** Clarifications on the issue have been added in the relevant sections (see page 10, lines 6-8; page 20, lines 20-24).

4. *The control group is described as being similar in age, nationality, civil status, and education level. However, it is not clear whether matching was done precisely or if this was achieved through random sampling. A more detailed explanation of the matching process would strengthen the methodological rigor of this section. For example, were controls individually matched to patients?*

**Reply:** Clarifications on the issue have been added in the relevant section (see page 7, lines 16-20).

5. *The exclusion criteria are well-detailed, but it would be beneficial to explain whether any additional steps were taken to control for confounding factors such as BMI, physical activity levels, or psychosocial stress, as these can influence cardiovascular and pain-related outcomes. Please update study limitations if needed.*

**Reply:** The issue is now addressed in greater detail in the relevant section (see our response to the previous comment regarding weight). Additional information is provided in Table 1, which includes demographic measures such as regular physical activity and affective conditions, as well as in Figures 1-7, showing effects before and after adjustments for the respective covariates.

Additionally, a second session was incorporated into the original CPAST protocol to enhance the robustness of the test results, mitigating the impact of arousal effects from various between- and within-subject confounding factors that change over time, including adaptation to psychosocial stress and other influences that could affect cardiovascular responses during the clino-orthostatic procedures. This additional information was included in the method section (page 11, lines 22-25, page 12, line 1).

6. *A brief mention of how the sample size was determined or whether a power analysis was conducted would strengthen the validity of the study. Was the sample size based on previous studies in fibromyalgia, or calculated to ensure sufficient statistical power for the planned analyses?*

**Reply:** The issue is addressed in the relevant section (see page 13, lines 19-22).

7. *It would be useful to specify if the tests were performed in the morning, afternoon, or at random times. Time of day can influence cardiovascular metrics, and standardizing test timing would reduce variability*

**Reply:** Clarifications on the issue have been added in the relevant section (see page 10, lines 8-11).

8. *While participants were instructed to refrain from alcohol, smoking, caffeine, and exercise for three hours, three hours may be too short a time to eliminate the potential effects of these factors, especially caffeine. It would be beneficial to clarify whether this time period was based on previous studies or other justifications. Please update study limitations if needed.*

**Reply:** Clarifications on the issue have been added in the relevant section (see page 10, lines 4-6).

9. *Please mention whether potential confounders like sleep quality or daily pain fluctuations were considered when scheduling participants' appointments. Since FM patients often experience fluctuating symptoms, were steps taken to control for the time of day or participant-specific variability in pain levels? This could improve the consistency of physiological and psychological measurements.*

**Reply:** Clarifications on the issue have been added in the relevant section (see page 7, lines 10-13).

10. *The electrode placements for ECG and ICG are described in good detail, but providing a visual or diagram in the supplemental materials would help, particularly for those less familiar with these measurement techniques*

**Reply:** The requested figures have been added as S Materials.IX with reference to them at a respective section (page 12, lines 11 and 13-14).

11. *It would be useful to elaborate on the clinical implications of these findings. For example, how might these differences in SV response influence the management of FM patients in clinical settings?*

**Reply:** This issue has been addressed in short in the 'Perspectives' subsection of the Discussion (pages 54-55) and further elaborated in details in S Materials.VII.

12. *The limitations of the study are well-addressed, but it could be useful to discuss any potential strategies for mitigating these limitations in future research.*

**Reply:** The respective approaches and plans to address the limitations have been briefly included in the subsection if previously omitted (page 52, lines 25-26; page 53, lines 1, 19-20, and 24-25; page 54, lines 9-12, 19-21; page 55, lines 6-13, 18-24). Most limitations discussed in the subsection are primarily methodological. Some of them, like the need for greater sex, age, and ethnic diversity, are suggested to be addressed in future studies. Others, such as noise during the short transition periods between lying and standing, cannot yet be fully resolved with the current acquisition techniques.

13. *Which cardiovascular metric you found most affected by pain? In total? In lying? And in Standing? Why do you think that metric is most affected?*

**Reply:** We partially addressed this point by discussing in the perspectives subsection the prompt and rapid vasodilation, rise of HR and CO in response to orthostatic challenges as the most reliable pain-o-metric indicators and as more perspective indicators for mobile health applications (page 56, line 11-15). This issue, in relation to these and some other indicators, has now been further addressed on pages 57 (lines 17-26) and 58 (lines 1-6).

14. *Authors mentioned “Data supporting this study are not currently publicly available due to its farther use by the research team.” At least, the data used for the plots in this study should be presented in Supplement I believe.*

**Reply:** All plots in the manuscript were generated using an adaptive data-driven technique from the raw dataset and therefore cannot be attributed to a separate dataset. Additional information regarding the availability of the dataset, with independent access from the authors, has been included in the appropriate section of the manuscript in response to the editorial request.

15. *Could you explicitly indicate what is the novelty of this paper? Has any other study in the literature shown this novelty before?*

**Reply:** This point was addressed in the introduction section at page 4 (lines 22-24) and now additionally is emphasized in the conclusion section (page 58, lines 9-17).

16. *Please highlight the potential clinical significance of the key differences found in this study?*

**Reply:** This issue appears to be closely related to the reviewer's comment 9. It is now further addressed in the 'Perspectives' subsection of the Discussion (pages 56-57), with additional detailed elaboration provided in S Materials.VII.

Sincerely yours, Dmitry M. Davydov, corresponding author.
